# ATDD: Multi-lingual dataset for auto-tune detection in music recordings

**DOI:** 10.1016/j.dib.2025.112446

**Published:** 2026-01-07

**Authors:** Mahyar Gohari, Paolo Bestagini, Sergio Benini, Nicola Adami

**Affiliations:** a Department of Information Engineering, CNIT - University of Brescia, Italy; b Department of Electronics, Information and Bioengineering, Polytechnic University of Milan, Italy

**Keywords:** Music information retrieval, Multimedia forensics, Pitch correction, Machine learning, Audio signal processing

## Abstract

This study introduces a novel multilingual dataset designed to distinguish auto-tuned musical compositions from authentic recordings, addressing a significant gap in existing resources. The dataset encompasses songs in English, Mandarin, and Japanese, ensuring a diverse representation of linguistic contexts. The data collection process began with aggregating diverse datasets from the Music Information Retrieval domain, incorporating tracks from the three specified languages to capture a wide range of musical styles and recording qualities. Each audio file was subsequently standardized into 10-second intervals with the sample rate of 16 kHz to facilitate manageable analysis. For the creation of auto-tuned samples, pitch correction was implemented using the probabilistic YIN (PYIN) algorithm for accurate pitch detection, followed by transposition via the pitch-synchronized overlap and add (PSOLA) technique. To emulate realistic auto-tuning scenarios, pitch correction was randomly applied to portions of each 10-second segment, ensuring variability and realism in the dataset, which makes it suitable for training robust detection models. Additionally, time-domain labels indicating the exact locations of pitch correction within each segment were generated, providing precise annotations crucial for developing accurate detection algorithms. The resulting multilingual dataset comprises a comprehensive collection of both auto-tuned and authentic musical segments across English, Mandarin, and Japanese languages, each annotated with detailed information about pitch correction applications. This rich annotation allows for nuanced analysis and supports various research applications, while the dataset's structure and thorough documentation of its creation process make it a valuable resource for researchers in music analysis, machine learning, and audio signal processing.

Specifications Table

**Table:** 

Subject	Computer Science / Signal Processing
Specific subject area	Detection and Localization of auto-tuned singing voices in music recordings
Type of data	(.wav) Raw, Processed (.json) Ground truth
Data collection	The dataset is curated by applying auto-tuning on eight open-source datasets in the literature. All the audio tracks are normalized to have an equal length of 10 seconds and resampled to 16 KHz.
Data source location	Department of Information Engineering, University of Brescia, Via Branze, 38, 25133, Brescia, Italy
Data accessibility	Repository name: Zenodo Data identification number: 10.5281/zenodo.14621005 Direct URL to data: https://doi.org/10.5281/zenodo.14621005 Direct URL to software: https://github.com/mahyargm/Auto-Tune-Detection
Related research article	M. Gohari, P. Bestagini, S. Benini and N. Adami, ``Spectrogram-Based Detection of Auto-Tuned Vocals in Music Recordings,'' 2024 IEEE International Workshop on Information Forensics and Security (WIFS), Rome, Italy, 2024, pp. 1-6, doi:10.1109/WIFS61860.2024.10810675.

## 1 Value of the Data

• This dataset serves as a valuable resource for developing and testing models to detect and locate auto-tuned vocals within song tracks. It provides researchers with the opportunity to create, assess, and benchmark new approaches for identifying and analyzing auto-tuning effects in musical recordings.
• This dataset represents an enhanced version of the dataset initially introduced in [1], now featuring a larger collection of multi-lingual songs along with strong labels that precisely mark the time stamps where auto-tuning is applied. Notably, auto-tuning is applied partially within tracks rather than uniformly across entire segments, thereby reflecting real-world practice and providing a stronger, more varied ground truth for learning-based methods. This extension provides a more comprehensive resource for training and validating auto-tune detection models.
• The code used to create this dataset has been published, enabling researchers to recreate the dataset and extend it using their own collection of songs, thereby facilitating the development of larger and more comprehensive datasets.

## 2 Background

As auto-tuning continues to play a significant role in contemporary music production, there is a necessary need to develop algorithms capable of identifying instances of auto-tuning in musical recordings. The motivation behind compiling this dataset stemmed from the need within the music information retrieval (MIR) community for standardized datasets to facilitate research on auto-tune detection methods.

This work extends the dataset we employed in our previous study [1], significantly enhancing its scope by increasing the number of songs, diversifying the languages represented, and incorporating strong labels that mark precise time stamps where auto-tuning is applied. The original dataset laid the groundwork for research in this area, but its scope was limited in terms of language diversity and label precision. In response, we expanded and enriched the dataset to provide a more comprehensive resource for researchers and developers.

This dataset aims to serve as a valuable resource for building, training, and evaluating machine learning models and algorithms for Auto-Tune detection. Additionally, to promote transparency and reproducibility, we provide the code used to create the dataset, allowing other researchers to extend it with their own music collections.

## 3 Data Description

The proposed dataset contains 21,249 isolated songs and singing voice segments sourced from various datasets in the MIR field to ensure diversity in styles, genres, and languages. Each song or singing voice in our dataset contains an auto-tuned version of themselves. Table 1 summarizes the overview of the proposed dataset. For practical use, we recommend further splitting the training set into training and validation subsets with a proportion of 90% to 10%, respectively.

**Table 1:** Overview of the dataset.

Source dataset		Number of 10s segments		
Name	Language	Training partition	Test Partition	Total
**MusDB18**	English	1,755	953	2,708
**VocalSet**	English (vowels)	3,613	-	3,613
**Opencpop**	Mandarin	1,881	-	1,881
**M4Singer**	Mandarin	5,645	5,034	10,679
**Ofuton-P**	Japanse	324	-	324
**Oniku Kurumi**	Japanse	417	-	417
**Kiritan**	Japanse	381	-	381
**JVS-MuSiC**	Japanse	1,246	-	1,246

The ground truth, relative paths, and additional details of the audio files are recorded in two separate JSON files, one for the training partition and one for the test partition. Each audio file in the dataset has an associated JSON metadata entry with the fields shown in Table 2.

**Table 2:** The structure of audio files' metadata.

Field	Description
Audio_path	Relative path to the audio file.
Source_dataset	Primary dataset of the original song or singing voice.
Partition	The partition that the segment belongs to. (Training or Test).
Auto-tuning_label	Includes the start time, end time, and duration of auto-tuning.
Song_name	Identifier for the song.
Singer(VocalSet)	Identifier of the singer if the song belongs to VocalSet.

## 4 Experimental Design, Materials and Methods

In this section, we initially present the primary datasets employed in this study. Subsequently, we explain the auto-tuning procedure utilized for the generation of Auto-Tuned songs and vocal tracks within the dataset. Finally, we provide a detailed, step-by-step procedure of the dataset creation process.

### 4.1 Primary datasets

In this study, we utilize 8 primary datasets to create the final dataset:

**VocalSet** [2]: The VocalSet comprises 10.1 hours of monophonic audio recordings, with durations ranging from 1.56 to 81.21 seconds and an average duration of 8.76 seconds. The recordings feature performances by professional singers and were chosen specifically for their diverse range of vocal techniques, demonstrated by 20 different singers. The application of auto-tuning to these vocals facilitates a comprehensive exploration and analysis of its effects.

**MusDB18** [3]: MusDB18, also referred to as the Music Source Separation Dataset, comprises 150 full-length music tracks representing various genres such as pop, rock, electronic, jazz, and more. It is divided into a training set containing 100 tracks and a testing set containing 50 tracks. Musdb18 is widely recognized as a benchmark dataset in the field of music source separation.

**Opencpop** [4]: A publicly available high-quality Mandarin singing corpus designed for singing voice synthesis (SVS) systems. It comprises 100 unique Mandarin songs recorded by a professional female singer in a studio environment, totaling approximately 5.2 hours of audio.

**M4Singer** [5]: A comprehensive Mandarin singing corpus featuring recordings from 20 professional singers, covering various styles and all four SATB (soprano, alto, tenor, bass) voice types. It encompasses 700 Chinese pop songs.

**Ofuton-P** [6]: A Japanese singing voice database featuring recordings from a single male singer, utilized in research and development of singing voice synthesis systems.

**Oniku Kurumi** [7]: Another Japanese singing voice database comprising recordings from a single female singer.

**Kiritan** [8]: Also known as the Tohoku Kiritan Singing Database, this Japanese singing voice corpus features recordings from a single female singer. It includes 50 Japanese pop songs totaling approximately 57 minutes of studio-recorded vocals.

**JVS-MuSiC** [9]: This dataset features recordings from 100 Japanese singers, each performing ``Katatsumuri,'' a traditional children's song, along with an additional unique song per singer.

### 4.2 Pre-processing process

To create a uniform dataset for analysis, we standardized all audio files from the primary datasets into 10-second segments with a sampling rate of 16 kHz. For every dataset except VocalSet, we divided each song into consecutive 10-second clips and discarded any remaining audio shorter than 10 seconds. This approach ensured consistent segment lengths and simplified subsequent data processing.

In contrast, VocalSet features a variety of standard and extended vocal techniques demonstrated on all five vowels, with most recordings lasting less than 10 seconds. These recordings are not typical singing voices but rather specialized vocal performances. Therefore, when standardizing the dataset into 10-second segments, we employed a different processing strategy for VocalSet: trimming longer recordings and applying zero-padding to shorter ones. This approach preserves the unique characteristics of VocalSet’s specialized vocal techniques while ensuring uniform segment lengths across the entire dataset.

Finally, we filtered out segments with low energy content to discard segments devoid of vocal elements. To do so, we defined

(1) $$E_{x}=\sum_{n=0}^{N-1}{\left|x\left[n\right]\right|}^{2}$$

which represents the energy of a N-samples floating-point discrete-time signal x, normalized between -1 and +1. We filtered out all segments with energy below 10, which corresponds to approximately less than 0.002 % of the maximum energy. This step effectively removes segments that do not contain any vocals from the dataset.

### 4.3 Auto-tuning process

We first randomly selected a portion of each audio segment, ranging from 20 % to 80 % of its total length, and applied auto-tuning, specifically to this portion, following the methodology described in [1]. The process begins with pitch tracking, which involves determining the pitch at each time stamp—a fundamental task in Digital Signal Processing (DSP) for audio signals. Among the available methods, the probabilistic YIN (PYIN) algorithm [10] is particularly notable and widely recognized. PYIN builds upon the YIN algorithm [11], which estimates pitch by analyzing the autocorrelation function of the time-domain signal and refining these estimates. PYIN enhances YIN by incorporating a Hidden Markov Model, thereby improving the accuracy of pitch tracking.

After identifying the pitches, we adjusted them to the nearest MIDI note using the pitch-synchronized overlap and add (PSOLA) technique [12]. By leveraging PSOLA, the pitch modification process maintains synchronization and coherence across audio segments, ensuring smooth transitions between notes.

### 4.4 Dataset creation process

**Fig. 1:**
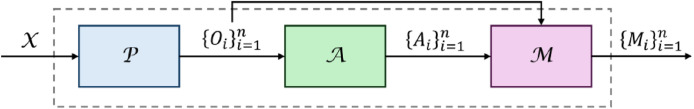
Dataset creation pipeline.

The dataset creation pipeline is illustrated in Fig. 1 using the three main building blocks: **Pre-processing (P), Auto-tuning (A),** and **Mixing (M).**

Initially, each isolated singing voice from the primary datasets undergoes segmentation in the pre-processing block (P), where each isolated singing voice X is divided into uniform 10-second clips, producing the pre-processed outputs ${\left\{O_{i}\right\}}_{i=1}^{n}$. These segments are then processed in the auto-tuning block (A), where pitch correction is applied to generate auto-tuned versions ${\left\{A_{i}\right\}}_{i=1}^{n}$. The Pre-processing and Auto-tuning processes are described in detail in 4.2, 4.3, respectively.

For tracks originating from the MusDB18 dataset, an additional mixing step is performed where the auto-tuned singing voices are recombined with their corresponding instrumental accompaniments to create auto-tuned mixed songs ${\left\{M_{i}\right\}}_{i=1}^{n}$. This final mixing ensures that the dataset includes auto-tuned mixed songs, which are more challenging for detection algorithms due to the complexity introduced by the background music. As a result, the final dataset comprises both isolated auto-tuned vocal segments and mixed auto-tuned songs, providing a versatile and demanding resource for advancing research in audio processing and auto-tune detection.

**Fig. 2:**
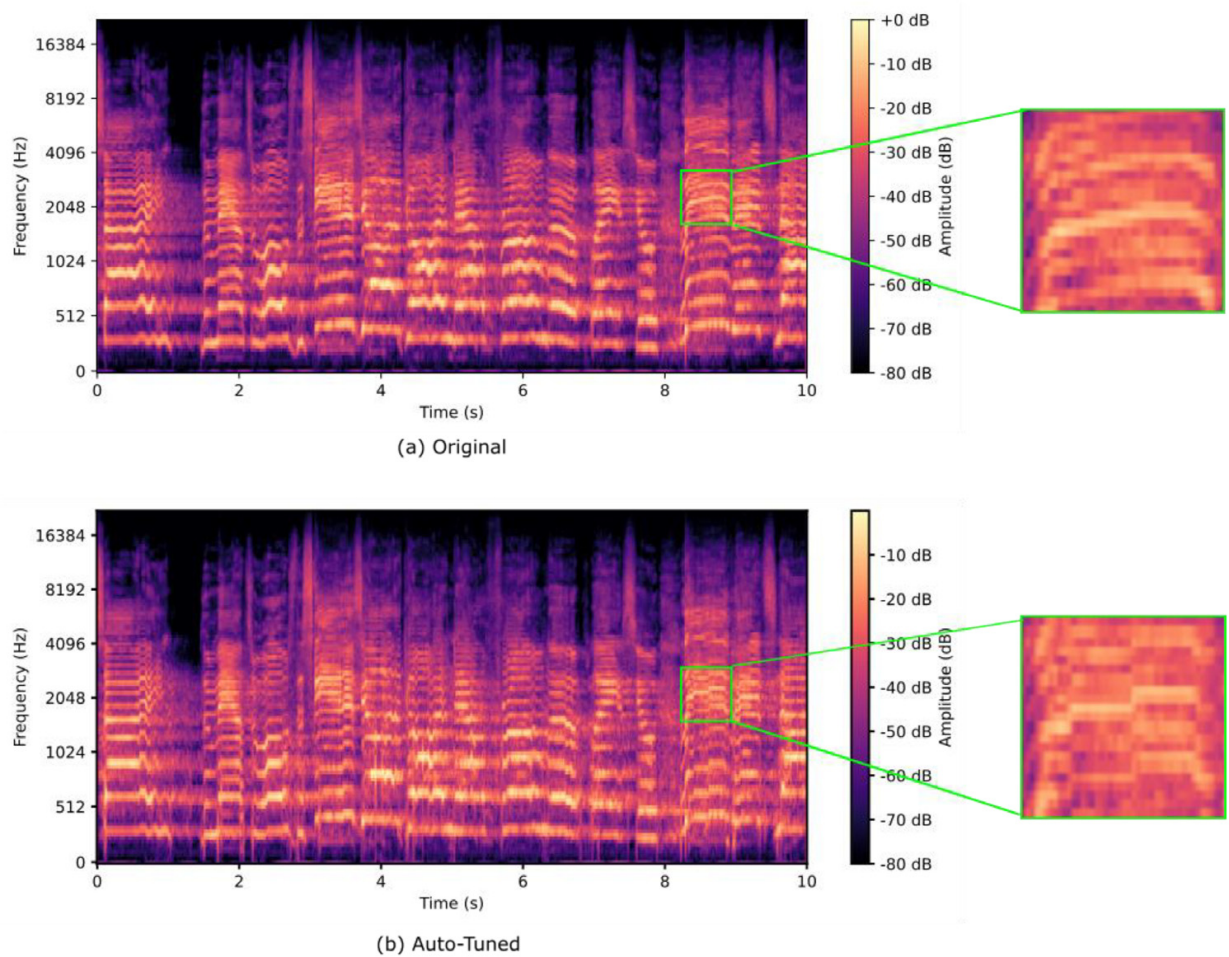
Mel-spectrogram representation of an audio segment and its corresponding auto-tuned version [1].

Fig. 2 compares a 10-second mel-spectrogram of an original audio file in our dataset with its corresponding Auto-Tuned version, illustrating the effectiveness of the pitch correction process.

## Limitations

While our study marks a significant step, it is essential to acknowledge certain limitations. Notably, the absence of manually and professionally Auto-Tuned songs in our dataset represents a constraint. Although we successfully replicated auto-tuning behavior using the employed method, the inclusion of manually and professionally Auto-Tuned samples would have provided a more comprehensive evaluation of models trained on this dataset.

## Ethics Statement

The authors have read and follow the ethical requirements for publication in Data in Brief and confirming that the current work does not involve human subjects, animal experiments, or any data collected from social media platforms.

## CRediT Author Statement

**Mahyar Gohari:** Writing – original draft, Software, Data curation, Visualization, Formal analysis, Investigation. **Paolo Bestagini, Sergio Benini, Nicola Adami:** Conceptualization, Methodology, Supervision, Project administration, Formal analysis, Investigation, Writing – review & editing.

## Data Availability

ZenodoATDD: Multi-lingual Dataset for Auto-Tune Detection in Music Recordings (Original data)
